# Local Periarticular Infiltration with Dexmedetomidine Results in Superior Patient Well-Being after Total Knee Arthroplasty Compared with Peripheral Nerve Blocks: A Randomized Controlled Clinical Trial with a Follow-Up of Two Years

**DOI:** 10.3390/jcm12155088

**Published:** 2023-08-02

**Authors:** Patrick Reinbacher, Gregor A. Schittek, Alexander Draschl, Andrzej Hecker, Andreas Leithner, Sebastian Martin Klim, Kevin Brunnader, Amir Koutp, Georg Hauer, Patrick Sadoghi

**Affiliations:** 1 Department of Orthopaedics & Traumatology, Medical University of Graz, 8036 Graz, Austria; patrick.reinbacher@medunigraz.at (P.R.); patrick.sadoghi@medunigraz.at (P.S.); 2 Department of Anesthesiology and Intensive Care Medicine, Medical University of Graz, 8036 Graz, Austria; 3 Division of Plastic, Aesthetic and Reconstructive Surgery, Department of Surgery, Medical University of Graz, 8036 Graz, Austria; 4 COREMED—Centre for Regenerative Medicine and Precision Medicine, Joanneum Research Forschungsgesellschaft mbH, 8010 Graz, Austria

**Keywords:** pain management, total knee arthroplasty, well-being, dexmedetomidine, local infiltration analgesia, peripheral nerve block

## Abstract

Background: This study aimed to compare local periarticular infiltration (LIA) with ultra-sound guided regional anesthesia (USRA) with ropivacaine and dexmedetomidine as an additive agent in primary total knee arthroplasty (TKA). Methods: Fifty patients were randomized into two groups in a 1:1 ratio. Patients in the LIA group received local periarticular infiltration into the knee joint. The USRA group received two single-shot USRA blocks. Functional outcomes and satisfaction (range of movement, Knee Society Knee Score, Western Ontario and McMaster Universities Osteoarthritis Index, Oxford Knee Score, and Forgotten Joint Score), including well-being, were analyzed preoperatively and at five days, six weeks, and one and two years postoperatively. Results: Functional outcomes did not significantly differ between the two groups at six weeks and one and two years after the implementation of TKA. A moderate correlation was observed in the LIA group regarding well-being and pain on day five. Six weeks postoperatively, the LIA group showed significantly superior well-being but worse pain scores. No differences between the groups in well-being and functional outcomes could be observed one and two years postoperatively. Conclusion: Patients treated with LIA had superior postoperative well-being in the early postoperative phase of up to six weeks. Furthermore, LIA patients had similar functionality compared to patients treated with USRA but experienced significantly more pain six weeks postoperatively. LIA leads to improved short-term well-being, which is potentially beneficial for faster knee recovery. We believe that LIA benefits fast-track knee recovery with respect to improved short-term well-being, higher practicability, and faster application.

## 1. Introduction

Postoperative pain management is still developing [1] but is crucial for successful patient recovery, rehabilitation [2,3], satisfaction, and perioperative well-being [4,5,6,7,8,9]. Currently, no ideal analgesic protocol for total knee arthroplasty (TKA) performs best in all outcome measures, including well-being [10,11].

The optimization of analgesia in TKA patients plays an important role in postoperative clinical outcomes, as there is a strong association between postoperative pain, early recovery, and functionality [12]. Postoperative pain can impair recovery and hinder early rehabilitation [2,13]. As rehabilitation should start immediately after surgery, pain management in TKA should permit adequate knee movement with minimal pain and no motor blocking to accelerate early mobilization for better postoperative functionality of the affected knee [14].

In recent years, there has been a shift toward using multimodal analgesic regimens to aim at multiple pain pathways while reducing opioid consumption. Among these, multimodal pain regimens utilizing local infiltration analgesia (LIA), also known as the local periarticular infiltration anesthesia technique, and peripheral nerve blocks (PNB) have emerged to handle surgical pain and enable early postoperative mobilization [15,16,17].

Among peripheral nerve blocks (PNBs), the femoral, sciatic, and obturator nerves are the most common targets for postoperative analgesia by ultrasound-guided regional anesthesia (USRA) in TKA [18]. Furthermore, the combination of a femoral (FNB) and sciatic nerve block (SNB), which contributes to additional improved pain relief compared to FNB alone [19,20], is described by a recent meta-analysis as one of the best options when it comes to early pain relief [21].

However, although the combination of FNB and SNB shows excellent results in pain reduction, it is associated with reduced mobility from muscle weakness, which can prevent a rapid recovery from occurring [10,22]. This is one of the reasons that LIA has been the subject of increasing interest in recent years [23]. Besides its advantages regarding lower complication rates and reduced systemic toxicity, the application of LIA in TKA is simple and fast [15,18,24]. Moreover, the analgesic effect of both approaches is reported to be comparable, with no significant difference in the short-term, making LIA a feasible alternative to combined femoral and sciatic nerve blocks [25].

The use of perineural dexmedetomidine in combination with nerve blocks has emerged as a potentially promising approach to enhance the outcomes of regional anesthesia [26,27,28]. Several studies have reported positive results, including a prolonged block duration, effective postoperative pain relief, and increased patient satisfaction [26,27,28]. As a result, dexmedetomidine as an adjuvant has garnered attention for its potential to improve the overall patient experience during and after surgical procedures in different settings, including regional and epidural anesthesia and analgesia [29,30]. In the context of epidural analgesia and anesthesia, dexmedetomidine as an adjuvant has been subjected to a meta-analysis, confirming its general safety and tolerability. The findings suggest that dexmedetomidine can be used as a valuable adjuvant in epidural analgesia and anesthesia, providing additional benefits in terms of pain control and patient comfort [30].

Concerning regional anesthesia and analgesia, it has shown superiority over fentanyl in elective cesarean sections by increasing the time to first rescue analgesia and prolonging the duration of the sensory block [29]. Additionally, a study by Schittek et al. provided data on TKA patients who received USRA with FNB and SNB as well as LIA with dexmedetomidine as an adjuvant in both groups [18]. The authors observed significantly more pain in the USRA group than in the LIA group at rest and exercise one day after surgery, with no meaningful difference between the study groups until the sixth postoperative day [18]. Furthermore, they detected a longer-lasting opioid-sparing effect in both groups, which they attributed to the addition of dexmedetomidine.

Given these promising outcomes, dexmedetomidine has also been described as one of the most promising additive drugs in the field of regional anesthesia [31]. However, there is a lack of data regarding the impact on well-being and early functional outcomes when adding dexmedetomidine to the USRA approach with FNB and SNB or the LIA approach for TKA patients.

As LIA is a feasible alternative to USRA due to its ease of implementation and rapid placement in clinical practice, we examined these two concepts in TKA as part of this prospective randomized controlled study with a two-year follow-up. We focused on ambulation, postoperative well-being, and functional outcome scores after surgery.

This study aimed to compare local periarticular infiltration (LIA) with ultrasound-guided regional anesthesia (USRA) with ropivacaine and dexmedetomidine as an additive agent in primary total knee arthroplasty (TKA).

## 2. Materials and Methods

This randomized, controlled clinical trial (RCT) followed accepted ethical, scientific, and medical standards and was conducted in compliance with recognized international standards, including the principles of the Declaration of Helsinki. Informed consent was obtained from all the participants, and the study protocol was approved by the institutional Ethics Committee (32–239 ex 19/20) and registered with data safety authorities (study registry: ClinicalTrials.gov, NCT04697537).

### 2.1. Study Population

The study’s cohort was based on a previous study [18] that examined two novel analgesic regimens for TKA using dexmedetomidine additionally in LIA and USRA, focusing on opioid consumption, postoperative pain, and complications, but was terminated due to ethical considerations. With a minimum follow-up of two years in this study population, we aimed to gain new insights into the effects of the described analgesic regimens on patients’ clinical outcomes and well-being up to two years postoperatively. We included consecutive patients from February to April 2021. Adult patients with end-state osteoarthritis were included in the study. Every patient enrolled in the randomized, controlled clinical trial analysis study received an Attune TKA (DePuy Synthes, Warsaw, IN, USA) operated by the same senior surgeon. The Attune Knee system is a versatile implant system for TKA [32]. It was developed by DePuy Synthes was introduced to address concerns about anterior knee problems and high dissatisfaction rates (up to 21%) associated with the previous PFC Sigma TKA by DePuy Orthopaedics [33]. The system had a limited launch in 2011 and was formally launched in 2013 [32,34]. The new design features a femoral component with a gradually reduced radius, enhancing conformity with the polyethylene insert to allow gradual femoral rollback and greater mid-flexion stability; in addition, the marketing emphasizes the unique patellar system for improved tracking and bone coverage [35,36]. Moreover, the tibial base component integrates a central locking system, aiming to provide more secure fixation and reduce micromotion at the backside of the implant [37].

Patients were randomly assigned to the USRA or LIA group in a 1:1 ratio. A web-based randomization tool from the Institute for Medical Informatics, Statistics, and Documentation (https://www.randomizer.at, accessed on 27 November 2020, certified according to ISO-9001:2015) generated the random allocation sequence before the surgery. Patients and physicians were aware of the group assignments. In the LIA group, patients were given local infiltration analgesia from the surgeon at the end of TKA. In the USRA group, patients received two ultrasound-guided peripheral nerve blocks from their anesthesiologist immediately before anesthesia induction in the operating theater. Postoperatively, the patients followed a standardized rehabilitation protocol, which consisted of full weight bearing with crutches immediately after surgery and continuous passive motion (CPM) on the first postoperative day. The study adhered to the applicable CONSORT guidelines [38].

### 2.2. Local Infiltration Anesthesia Procedures and Regional Anesthesia

Patients in the LIA group received periarticular infiltration with 60 mL ropivacaine 0.5% and 1 mL dexmedetomidine (100 μg mL^−1^) around the knee joint, including the posterior capsule, to block distal nerve fibers. The volume LIA was distributed according to the surgeon’s choice. The infiltration was performed before positioning the liner and after the femoral and tibial components’ implantation. Before skin closure and the end of surgery, the infiltration procedure treated the knee joint capsule, posterior joint structures, periarticular soft tissue, and subcutaneous soft tissues.

According to the local standard operating procedure, both single-shot peripheral nerve blocks were conducted in the USRA group immediately before the induction of general anesthesia or spinal anesthesia. A 120-mm 22-gauge needle (Pajunk SonoplexStim; GmbH Medizintechnologie, Geistigen, Germany) was used under sterile conditions to perform the blocks. A linear ultrasound transducer (frequency 10 to 12 MHz) was used to visualize the target nerves, the needle, and the surrounding structures.

Approximately 1–3 cm before the sciatic nerve’s division into the common perineal and tibial nerves and at a safe distance from the popliteal fossa, the distal single-shot sciatic nerve was performed. The nerve block was performed in the supine position, with the foot resting on an elevated footrest. An ultrasound-guided in-line needle insertion technique was used for needle placement and control of local anesthetic spread. Perineurally, a mixture of 15 mL ropivacaine 0.5% and 0.5 mL dexmedetomidine (100 μg mL^−1^) was injected. To reduce patient discomfort during regional anesthesia, ultrasound-guided femoral nerve blockade with the simultaneous intravenous administration of remifentanil 20 was performed before anesthetic induction. Thus, patients were placed in the supine position to access the groin. Another mixture of 15 mL ropivacaine 0.5% and 0.5 mL dexmedetomidine (100 µg mL^−1^) was injected perineurally with an ultrasound-guided in-line needle insertion technique for proper needle placement. One senior anesthesiologist performed USRA.

### 2.3. Surgical Technique and Anesthetic Management

All TKA procedures were carried out by one senior knee surgeon using the same surgical technique via the medial parapatellar approach with no patella resurfacing, with an extension gap first flexion gap balanced system (Attune, DePuy Synthes, West Chesrer, PA, USA). Both the femoral and tibial components were cemented (Palacos R + G, Heraeus Medical, Wehrheim, Germany). Attending anesthesiologists were not limited in their clinical management of the patients, except that no peripheral nerve blocks were allowed in the LIA group.

### 2.4. Outcome Measurement

The endpoints for analysis were functional outcome parameters. The following questionnaires were used: Knee Society Knee Score (KSKS) and Knee Society Function Score (KSFS) [39], Western Ontario and McMaster Universities Osteoarthritis Index (WOMAC) [40], Oxford Knee Score (OKS) [41], Forgotten Joint Score (FJS) [42], and the English version of the “Evaluation du Vécu de I’Anesthésie LocoRégionale“ (EVAN-LR) [43]. In addition, the Anästhesiolgischer Nachbefragungsbogen (ANP) [44] has been validated to assess postoperative disturbances and satisfaction. The ANP was used to determine well-being. The range of motion (ROM) was measured with a double-armed goniometer. Patients were evaluated preoperatively and 5 days, 6 weeks, 12 months, and 24 months postoperatively.

### 2.5. Statistical Analysis

Data were reported as numbers of patients in percent, means (±SD) for parametric data or medians (25 to 75 percentiles [IQR]) for nonparametric data, and the Kolmogorov–Smirnov and Shapiro–Wilk tests were used for normal distribution testing. For univariate analyses of statistical significance, Fisher’s exact test or the Mann–Whitney test for nonparametric data were performed. Statistical significance was analyzed with a two-sided alpha of less than 5% as a significance level. Further analyses included rank correlation with Spearman’s ρ and logistic regression. Spearman correlations were performed to assess a possible correlation between the use of LIA and the items of the questionnaires (at rest and during exercise). For the logistic regression models for well-being, the covariates “type of anesthesia” (general anesthesia [binary]), “type of administration of local anesthetics “(LIA [binary]), and “sex” (binary) were adjusted. The well-being Likert scores with a threshold of good (two lowest disturbance scores) and bad (two highest scores) were dichotomized in this logistic regression analysis. A priori power analysis (Statistical Solutions Ltd. nQuery Advisor Version 8.4.1 2019; Cork, Ireland) regarding the endpoints well-being and clinical outcome was performed with a difference of 10% set for clinical relevance and revealed a number of n = 25 per group as sufficient, with a *p*-value < 0.05 and a power greater than 80%. Statistical significance was analyzed with a two-sided alpha of less than 5% as a significance level. Correlations were defined as weak when r = 0.10–0.29, moderate when r = 0.30–0.59, and strong when r > 0.59 (and vice versa for negative correlations).

## 3. Results

**Figure 1:**
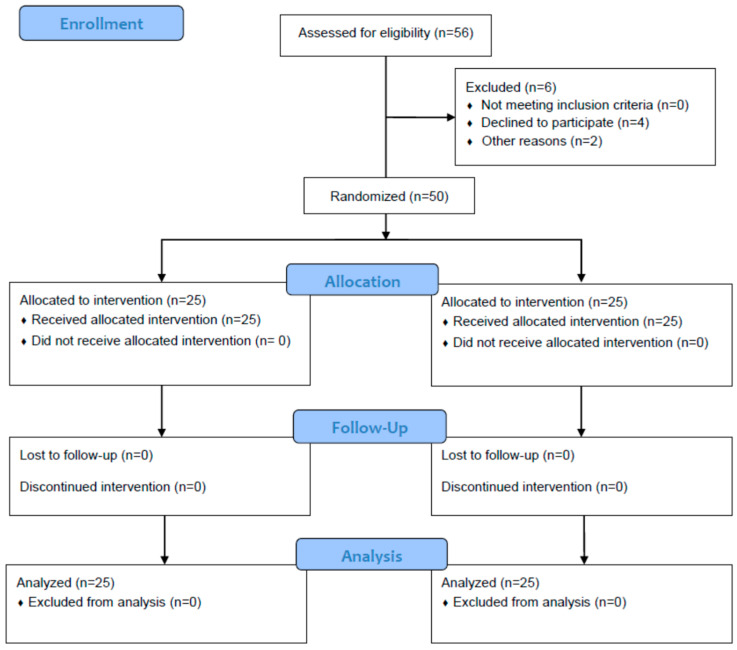
Study enrollment. Fifty-six consecutive patients were screened for eligibility. These patients were randomized into two groups. One group was given local periarticular infiltration anesthesia (LIA) into the knee capsule during surgery and the other was given two single-shot ultrasound-guided regional anesthesia (USRA) blocks.

Of 56 consecutive patients screened for eligibility (Figure 1), 50 were randomized and included in the final analysis. No dropouts and no complications associated with USRA or LIA were observed in this study. The characteristics of the patients did not differ but for the more frequently applied general anesthesia in the USRA group. Spinal anesthesia was more frequent in the LIA group (*p* = 0.037). No significant differences were observed between the two groups in baseline characteristics and demographics (Table 1).

### 3.1. Well-Being

The analysis of the questionnaires revealed that ten patients in the LIA group reported well-being, while only three did so in the USRA group, six weeks postoperatively (*p* = 0.024) (Table 1). No significant differences were found in well-being during the follow-up (*p* = 1.000).

### 3.2. Functional Outcome

Functional outcome scores differed only in KSKS pain on day 5, with higher pain scores in the LIA group (*p* = 0.011). Differences in KSKS pain were non-significant thereafter. Furthermore, there were no significant differences in the other clinical outcome scores after TKA with dexmedetomidine LIA or combined FNB and SNB in the short (day 5 and week 6) or long term (one and two years), as reported in Table 2.

### 3.3. Postoperative Improvement

Functional outcome scores differed only in KSKS pain on day 5, with higher pain scores in the LIA group (*p* = 0.011). Differences in KSKS pain were non-significant thereafter. Furthermore, there were no significant differences in the other clinical outcome scores after TKA with dexmedetomidine LIA or combined FNB and SNB in the short- (day 5 and week 6), long-term (one and two years), which is reported in Table 2. 

### 3.4. Rank Correlation and Logistic Regression Analyses

Regarding the observed correlations between LIA and the questionnaires, only well-being and KSKS pain five days after surgery (r = 0.401, r = 0.362, *p* < 0.01) were correlated moderately. When the well-being of patients was placed in a logistic regression model adjusted for LIA, sex, and type of anesthesia (spinal or general anesthesia), only the performance of LIA remained significant (Table 3). The comparison between the USRA and LIA groups regarding return to sex (*p* = 0.231), allodynia (*p* = 0.191), and hyperalgesia (*p* = 0.280) six weeks and one and two years after surgery showed no significant differences between groups.

## 4. Discussion

This study aimed to compare local periarticular infiltration (LIA) with ultrasound-guided regional anesthesia (USRA) with ropivacaine and dexmedetomidine as an additive agent in primary total knee arthroplasty (TKA).

The most important finding of our investigation was that patients reported significantly higher rates of well-being when LIA was performed than USRA, despite higher postoperative opioid requirements during the first 24 postoperative hours [18]. Although a higher rate of well-being was observed in the LIA group six weeks postoperatively, there were no differences between the two groups one and two years after TKA. Moreover, no differences in the long term could be observed concerning clinical outcomes, including pain.

This could mean that the sensory/motor block caused by USRA has a greater influence on early well-being than more intense pain and a greater need for opioids, as observed in our LIA group. We interpret this in light of the patients’ expectations, which certainly include postoperative pain more often than temporary motor paralysis for up to two days postoperatively, leading to the aforementioned results. This circumstance can probably be best explained by the brief and simplified definition of well-being, “… the state of feeling healthy and happy”, which can only be assessed subjectively [45].

It is known that general physical well-being affects satisfaction in patients following TKA [46]. Furthermore, psychological factors, such as tangible support, depression, dysfunctional coping, and low optimism, are associated with higher pain and inferior results in functionality as well as patient satisfaction after TKA [47]. Hence, we interpret this as growing evidence that well-being, including physical and mental components, appears to play a more important role than previously thought.

Kampitak et al. [48] assessed patient satisfaction in their study, in which LIA and an adductor canal block (ACB) were compared. Contradictory to our observed well-being scores, the patient satisfaction score of the LIA group was inferior to that of the USRA group; however, the difference was statistically non-significant. Kastelik et al. [49] presented comparable results in patient satisfaction and requirements for postoperative oral morphine equivalents during the hospital stay between LIA and single-shot SNB combined with ACB, which is different from our findings. Moreover, Uesugi et al. [50], comparing combined FNB and SNB with LIA, found no significant difference in satisfaction with analgesia up to 48 h after TKA. However, in the present RCT, we showed superior short-term well-being rates for LIA compared to USRA for the first time, although the LIA group experienced significantly more pain on day 5 after TKA. We see the greater well-being observed six weeks postoperatively as a psychological advantage with a potentially higher grade of motivation for rehabilitation, which could lead to improved knee recovery and overall satisfaction. Improvements in functional outcomes due to early mobilization [51,52,53] and the beneficial effects of LIA on functional recovery and pain control have been repeatedly described [54,55,56,57].

Regarding postoperative short-term functionality, our findings align with previous studies that evaluated patients who underwent TKA with regional anesthesia or LIA, showing no significant differences up to one year after surgery [13,58]. Fan et al. evaluated the KSKS function score up to one year after TKA in patients receiving either regional anesthesia with FNB or LIA [58]. In accordance with their results, we did not observe significant differences in short-term functionality up to one year post-TKA. Furthermore, the lack of statistically significant differences regarding postoperative short-term functionality observed in our study is consistent with the findings of Li et al. [13], who assessed patients undergoing TKA with regional anesthesia involving a combined ACB and lateral cutaneous femoral nerve block (LCFNB) versus LIA. Similar to our findings, they also did not observe a significant difference in the KSKS function score between the two groups at three months post-surgery, which is comparable with our findings six weeks to six months after surgery [13]. Hence, these results suggest that both regional anesthesia and LIA appear to be comparably effective in facilitating short-term functional recovery for patients following TKA. However, it is important to mention that, when comparing FNB with LIA, Yu et al. [57] observed significantly more falls in the FNB group during the hospital stay, potentially leading to anxiety and further hindering the early rehabilitation process [59,60].

A recent study compared the additional implementation of dexmedetomidine with ropivacaine in LIA and USRA (femoral nerve block and popliteal nerve block) and revealed a superior opioid-sparing effect in both groups, with USRA being superior to LIA when compared directly [18]. As with these findings, another study demonstrated that LIA provided better results in pain control in the early postoperative period than ACB after TKA, which was beneficial to early postoperative rehabilitation and added to patient satisfaction [56]. Aso et al. [61] described that performing LIA in addition to an FNB is an effective method for postoperative pain management after TKA. Lychagin et al. [24] compared LIA with combined FNB and SNB in TKA patients and found that the PNB only provided significantly better pain relief 4 h postoperatively, with no further significant differences in pain until the fifth day after surgery. The non-significant difference between both groups differed from our results, which showed significantly more pain on the fifth day after surgery in the LIA group.

For patient satisfaction, FNB combined with LIA was determined as the best option [38]. Studies comparing LIA (using liposomal bupivacaine (LB)) with FNB found that LIA resulted in a greater number of patients ambulating on the day of surgery and faster and better recovery of function, but similar pain relief in both groups [54,57,62,63]. Furthermore, Surdam et al. [62] showed a reduction in the LIA group’s average length of hospital stay (LOS). According to Spangehl et al. [64], LIA provides comparable pain relief to single-shot SNB combined with an indwelling femoral nerve catheter and results in a slightly reduced length of hospital stay.

The results of LIA and various types of USRA in terms of functional outcomes, postoperative pain, length of hospital stay, satisfaction, and opioid consumption are still controversial in the current literature [48,55,56,57,62,65], and it seems rather impossible to point out an intervention that performs the best in all outcome measures. Furthermore, the lack of consistency in functional outcomes may be attributable to the heterogeneity of the used agents and perioperative pain management, as well as differences in the implemented interventions in previous studies. This makes it challenging to determine whether LIA or USRA is superior for TKA in clinical practice regarding functional outcomes, early postoperative pain, and well-being.

This RCT observed that USRA and LIA influence patient well-being and early postoperative pain differently but show similar functional outcomes. We emphasize that the decision regarding whether to perform LIA or USRA should be sought individually, primarily depending on the medical indications, patient expectations, and perceptions, including the careful evaluation of individual risk factors and benefits for each patient, as well as the goals of the rehabilitation process after surgery. Our results suggest that determining the postoperative analgesic method of choice in TKA patients should also rely on whether analgesia (USRA) or motor function (LIA) is the priority, especially in the early postoperative period, to improve patient outcomes.

## 5. Limitations

We wish to underline that the discrepancy between spinal and general anesthesia, with more LIA patients having undergone spinal anesthesia, was a confounder within the data. Moreover, this study did not compare outcomes during the first four postoperative days, which would likely have provided additional valuable information for the comparison between the two groups, as the effects of the agents used typically disappeared after the first or second postoperative day. The observed differences among both groups (LIA vs. USRA) were based on the study’s small sample size, and the results should therefore be interpreted with caution. Based on the study’s limitations, we cannot suggest one method over the other as both approaches have advantages and disadvantages when it comes to well-being and pain in the early postoperative period.

## 6. Conclusions

Patients treated with LIA had superior postoperative well-being in the early postoperative phase of up to 6 weeks and had similar functionality in comparison to patients treated with USRA but experienced significantly more pain. LIA leads to improved short-term well-being, which is potentially beneficial for faster knee recovery, including the motivation for rehabilitation and physical therapy. Additionally, LIA has advantages in its practicability, as it is easier and faster to perform than USRA.

## Figures and Tables

**Table 1:** Patient characteristics, anesthesia, days of hospitalization, and well-being.

	USRA, N = 25	LIA, N = 25	*p*-Value
Age (years)	67.6 (±11.0)	68.6 (±10.2)	0.771
Female (%)	10 (40)	12 (48)	0.569
BMI (kg/m^2^)	27.8 [24.3 to 33.8]	28.4 [25.7 to 31.6]	0.734
ASA 1 (%)	1 (4)	0 (0)	
ASA 2 (%)	7 (28)	10 (40)	0.437
ASA 3 (%)	17 (68)	15 (60)	
General anesthesia (%)	11 (44)	5 (20)	0.037
Spinal anesthesia (%)	14 (56)	20 (80)	
Days of hospitalization	6.0 [6.0 to 7.0]	6.0 [6.0 to 7.0]	0.639
Well-being, N (%)			
Six weeks after surgery	No: 22 (88%) Yes: 3 (12%)	No: 15 (60%) Yes: 10 (40%)	0.024
12 months after surgery	No: 5 (11%) Yes: 42 (89%)	No: 4 (9%) Yes: 43 (91%)	1.000
24 months after surgery	No: 5 (11%) Yes: 42 (89%)	No: 4 (9%) Yes: 43 (91%)	1.000

LIA: local periarticular infiltration anesthesia technique; USRA: ultrasound-guided regional anesthesia; ASA: physical status classification system by the American Society of Anesthesiologists.

**Table 2:** Functional outcomes preoperatively and five days, six weeks, 12 months, and 24 months after primary TKA with dexmedetomidine LIA or USRA.

	USRA (n = 25)	LIA (n = 25)	*p*-Value
Range of Motion			
Preoperative	105 [100–115]	95 [85–115]	0.412
Five days postoperative	90 [90–100]	90 [90–100]	0.593
Six weeks after surgery	115 [110–120]	115 [110–120]	0.734
12 months after surgery	118 [90–145]	119 [100–145]	0.825
24 months after surgery	123 [100–150]	123 [100–150]	0.241
KSKS Pain			
Preoperative	59 [55–64]	55 [53–67]	0.464
Five days postoperative	65 [62–67]	75 [68–92]	0.011
Six weeks after surgery	92 [89–97]	90 [73–96]	0.907
12 months after surgery	96 [80–100]	95 [87–100]	0.497
24 months after surgery	98 [90–100]	98 [94–100]	0.189
KSKS Function			
Preoperative	50 [50–70]	50 [50–60]	0.565
Five days postoperative	20 [20–50]	30 [30–60]	0.257
Six weeks after surgery	50 [50–70]	50 [50–60]	0.757
12 months after surgery	83 [65–100]	84 [50–100]	0.659
24 months after surgery	93 [65–100]	93 [80–100]	0.643
WOMAC			
Preoperative	57.1 [54.2–63.4]	58.6 [55–62.3]	0.846
Five days postoperative	72.3 [65.9–78]	77.4 [75.1–80.3]	0.081
Six weeks after surgery	90.1 [85.3–94.1]	90.5 [90.3–95.3]	0.294
12 months after surgery	92.6 [86–100]	93.4 [86–100]	0.711
24 months after surgery	94.1 [90–100]	95.8 [90–100]	0.754
OKS			
Preoperative	19 [17–23]	16 [14–22]	0.255
Six weeks after surgery	31 [27–36]	31 [27–36]	0.712
12 months after surgery	38 [28–42]	37 [28–41]	0.862
24 months after surgery	43 [31–45]	43 [32–46]	0.897
FJS			
Six weeks after surgery	48 [47–51]	51 [49–53]	0.090
12 months after surgery	62 [48–75]	63 [49–78]	0.382
24 months after surgery	80 [60–92]	82 [58–94]	0.827

LIA: local infiltration anesthesia; USRA: ultrasound-guided regional anesthesia (combined femoral and sciatic nerve block); ROM: range of motion; KSKS: Knee Society Knee Score; WOMAC: Western Ontario and McMaster Universities Osteoarthritis Index; OKS: Oxford Knee Score; FJS: Forgotten Joint Score.

**Table 3:** Patient well-being in a logistic regression model adjusted for LIA, sex, and type of anesthesia.

	Exp (B)	95% CI for Exp (B)		*p*-Value
		Lower	Upper	
male sex	0.622	9 (50%)	30 (86%)	0.009
LIA	5.254	13 (72%)	27 (77%)	0.743
GA	0.748	4 (22%)	11 (31%)	0.539

Variables entered at step 1: male sex, local infiltration anesthesia (LIA), general anesthesia (GA). Exp (B): regression coefficient; CI: confidence interval.

## Data Availability

The datasets generated and/or analyzed during the current study are available from the corresponding author upon reasonable request.
